# Clinical characteristics and outcomes of cervical lymph node metastasis from unknown primary sites: a single institution’s 14-year experience

**DOI:** 10.1186/s40001-022-00957-9

**Published:** 2023-01-03

**Authors:** Mengqian Zhou, Yansheng Wu, Yue Wu, Hong Li, Beibei Ye, Kai Yue, Chao Jing, Yuansheng Duan, Xudong Wang

**Affiliations:** 1 Department of General Surgery, The Second Affiliated Hospital of Anhui Medical University, Hefei, 230601 Anhui China; 2 Department of Maxillofacial and Otorhinolaryngological Oncology, Tianjin Medical University Cancer Institute and Hospital, National Clinical Research Center for Cancer, Tianjin’s Key Laboratory of Cancer Prevention and Therapy, Tianjin’s Clinical Research Center for Cancer, Tianjin, 300060 China

**Keywords:** Cervical lymph node metastasis, Unknown primary lesion, Treatment, Survival

## Abstract

**Background:**

Cervical lymph node metastasis from unknown primary sites is a challenging clinical issue with a changing therapy model and unpredictable outcomes, which leads to the difficulty in selecting optimal treatments. Thus, it is valuable to analyze the clinical characteristics and outcomes of the patients who receive different management styles.

**Methods:**

All patients with cervical lymph node metastasis from unknown primary sites were reviewed and no primary lesions were found. In addition, this work was funded by the Clinical Trial Fund Project of Tianjin Medical University Cancer Institute and Hospital (No. C1716). Specifically, we used univariate, multiple regression analysis to evaluate the factors associated with prognosis.

**Results:**

365 patients met the inclusion criteria, and the 2- and 5-year survival rates were 77.0% and 33.4%, respectively, with a median survival of 45 months. Gender, age, pathological type, nodal status, and necessary cervical lymph node dissection affected locoregional control. Distant metastasis was common in individuals with a pathological type of adenocarcinoma, poor differentiation, and advanced nodal status. Furthermore, patients who received induction chemotherapy had a better prognosis than those treated with postoperative chemotherapy. Multiple regression analysis showed that pathological grade, treatment models, and distant metastasis were associated with overall survival (OS) and progression-free survival (PFS). In addition, local recurrence exerted a significant influence on OS. Induction chemotherapy and postsurgical radiotherapy seemed to improve the prognosis of patients at the advanced stage compared with simple surgery and postsurgical chemotherapy.

**Conclusions:**

Pathological grade, treatment models, and distant metastasis were independent risk factors for prognosis. Induction chemotherapy or postoperative radiotherapy benefited patients at the advanced stage, and patients with adenocarcinoma, poor differentiation, and advanced nodal status should undergo induction chemotherapy in light of the increased risk of distant metastasis.

## Introduction

Cervical lymph node metastasis from unknown primary sites refers to cervical malignant tumors without a primary lesion based on comprehensive examinations, which account for approximately 1–4% of head and neck carcinoma [1, 2]. There is growing evidence that the incidence of such disease has reduced in recent years, probably due to improved imaging modalities and detection methods which make it easier to detect incidental tumors [2]. In relation to the low incidence of carcinoma from unknown primary sites, previous studies have only conducted retrospective analyses with small sample sizes. There is a dearth of adequate participants, randomized clinical trials, and prospective studies [3]. Furthermore, no consensus has been achieved in terms of optimal diagnostic algorithm and treatment policy. Hence, investigators should rely on retrospective analyses to understand the disease in depth.

The National Comprehensive Cancer Network guidelines declare that suspicious sites should be treated in light of lymph node drainage patterns and subsequent local failure. Patients have excellent disease control and longer survival for N2–N3 cancers after combined-modality treatment with intensive chemoradiotherapy [4], while Fakhrian et al. did not find that comprehensive radiotherapy or concomitant radiochemotherapy have better outcomes when compared with less aggressive treatments [5]. Both chemotherapy and concurrent radiotherapy have become integral parts of the standard non-surgical treatments for advanced head and neck cancer; however, the proper role of chemotherapy in this disease remains controversial. There is a lack of literature regarding the management of combined-modality therapy, mainly because of the rarity of this presentation and optimum therapy for advanced nodal status, particularly the use of induction chemotherapy [6, 7]. Further studies about the role of concomitant or induction chemotherapy in patients with cervical lymph node metastasis from unknown primary sites are recommended.

Repeated inspections and high financial costs are distressing for patients. An in-depth analysis of the patient-specific risk factors could help to evaluate outcomes and guide personalized treatment regimens. In this retrospective study, we aimed to explore the clinical characteristics associated with prognosis and further establish appropriate treatments for improving outcomes.

## Methods

### Study cohort

We conducted a single-center retrospective review of patients diagnosed with cervical metastases in 2006–2020 at Tianjin Medical University Cancer Institute and Hospital. The inclusion criteria were: (1) patients with a histopathological diagnosis of cervical lymph node metastasis, (2) without a history of previous carcinoma, and (3) no primary lesions found during treatment or follow-up. The clinical and pathological characteristics are shown in Table 1.

**Table 1:** Patient characteristics involved in the study

Features	*N* = 365(%)
Gender	
Male	231 (63.3)
Female	134 (36.7)
Age	
≤ 60 years	205 (56.2)
> 60 years	160 (43.8)
Smoking	
None	224 (61.3)
No more than 20 years and 20/day	40 (11.0)
Above 20 years or 20/day	101 (27.7)
Alcohol	
None	300 (82.2)
No more than 20 years and 250 g/day	39 (10.7)
Above 20 years or above 250g/d	26 (7.1)
Lymph node level	
II/III	226 (61.9)
IV	139 (38.1)
Pathological type	
Squamous cell carcinoma	192 (52.6)
Adenocarcinoma	118 (32.3)
Other types	55 (15.1)
Pathological grade	
Highly differentiated	204 (55.9)
Moderately differentiated	9 (2.5)
Poorly differentiated	152 (41.6)
Involved nodal stage	
N1	38 (10.4)
N2a	22 (6.0)
N2b	185 (50.7)
N2c	110 (30.1)
N3	10 (2.7)
Initial treatment	
No treatment	54 (14.8)
Chemotherapy	91 (24.9)
Surgery	192 (52.6)
Radiotherapy	28 (7.7)

### Diagnostic work-up

Pretreatment examination included endoscopic examination of suspicious mucosal primary sites, chest scan, computed tomography (CT) scan, magnetic resonance imaging (MRI) of the neck and chest, or fluorodeoxyglucose–positron emission tomography (PET–CT). Three pathologists made the histopathological diagnosis according to the tumor classification of the World Health Organization.

### Treatment technique

Treatments included surgery, radiotherapy, chemotherapy, and multimodal treatments. For patients with N1 cancer, simple surgery or radiotherapy was recommended. Meanwhile, surgery and subsequent radiotherapy or chemotherapy were considered in patients with N2–3 cancer. If the neck mass was decreased in size after induction chemotherapy, patients with advanced disease would undergo radiotherapy; otherwise, patients with residual tumors were offered surgical options.

The surgical procedure consisted of regional mass resection, selective neck dissection, and modified radical neck dissection. Patients at the early clinical stage underwent simple surgery, while those at the advanced stage underwent further postoperative therapy.

The irradiated volume, dose, and fractional pattern were determined according to the extent of nodal involvement, presence of risk factors, and comprehensive consideration of the radiation oncologist. Furthermore, 49 patients underwent local irradiation with a dose of 50–54 Gy. Four patients had curative therapy, four were treated with chemotherapy, and 14 underwent radiotherapy after surgery. The remaining 27 patients were treated with chemotherapy and subsequent irradiation.

As an auxiliary measure, chemotherapy was usually combined with surgery and radiotherapy. Thirty-seven (24%) patients were treated with induction chemotherapy, 10 patients underwent surgery, and 27 (73%) underwent radiation to the neck. These treatments included TPF regimen (docetaxel on day 1, nedaplatin on days 2–3, and tegafur on days 2–6) every 3 weeks.

### Follow-up

Patients were followed-up primarily through telephone calls, letters, and outpatient reviews. As of June 2020, the follow-up period was in the range of 0.5–120 months. During the follow-up visit, all patients underwent the following examinations: physical examination, endoscopy, ultrasonography, and radiological examinations (MRI, CT scan, and bone scan). Any cause of death during follow-up was considered to be an end-point event. OS was considered as the primary endpoint and PFS was identified to be the secondary endpoint. OS was calculated from the date of initial diagnosis to the end-point event or last follow-up. PFS was measured from the date of initial diagnosis to the date of recurrence or progression.

### Statistical analysis

IBM SPSS 23.0 for Windows was used for the statistical analysis. The Mann–Whitney *U* and chi-square tests were used to analyze ordinal and categorical variables, respectively. Analysis of time-to-event curves was conducted using Kaplan–Meier survival curves and the log-rank test to assess the survival ratio difference. Furthermore, the Cox proportional hazards model was used to evaluate prognostic factors. All statistical tests were two-sided and *P* < 0.05 was considered statistically significant.

## Results

### Patient characteristics

The median age of all patients was 58 (range: 18–79) years; most patients were men (63.3%). Neck metastases were localized in levels II and III in 226 patients and level IV in the remaining 139 (38.1%) patients. The most common pathological type was squamous cell carcinoma in 192 (52.6%), followed by adenocarcinoma in 118 (32.3%), and other types in 55 (15.1%) patients. In addition, patients had the following nodal status: N1 (*n* = 38), N2a (*n* = 22), N2b (*n* = 185), N2c (*n* = 110), and N3 (*n* = 10) (Table 1).

The initial treatment was grouped into four types: chemotherapy, surgery, radiotherapy, and no treatment. A total of 192 (52.6%) patients underwent neck dissection in the form of mass dissection (15.1%), modified radical neck dissection (31.8%), and selective neck dissection (53.1%) at the early stage. Ninety-one patients received chemotherapy followed by surgery (10/91) or radiotherapy (27/91), and the remainder (*n* = 54) received simple chemotherapy. Among the 28 patients who received radiotherapy, eight (28.6%) patients underwent radiotherapy without neck dissection, of whom seven were considered inoperable. The other patients were treated with chemotherapy or surgery. Moreover, 54 patients initially refused any treatments after being diagnosed with cervical lymph node metastasis from unknown primary sites in this center. Among these patients, 21 had undergone simple surgery, 25 had been treated with postoperative radiotherapy, and eight had not received therapy in other hospitals. In general, 5.5% (20/365) of patients had neck failure and 81 patients presented with distant metastasis, of whom 73 (90%) were at N2b stage.

### Development of disease

In total, 5.5% (20/365) of patients presented with locoregional recurrence, 14 patients with recurrence in the affected neck side, and 6 patients with recurrence in the contralateral neck. The recurrence time ranged from 0.5 to 84 months, with a median recurrence time of 11 months. Gender, age, pathological type, nodal status, and surgery were important factors influencing the locoregional recurrence (Table 2). Gender (male), age (≤ 60 years), squamous cell carcinoma, N3, and no surgery significantly correlated with local recurrence. Notably, although patients with squamous cell carcinoma were at a higher risk of regional recurrence, the location of lymph node metastasis was unlikely to significantly affect local control.

**Table 2:** Local recurrence analysis of patients

Features	Number	Local recurrence (%)	*χ*^2^	*P*
Gender				
Male	231	19 (8.2)		
Female	134	1 (0.7)	9.159	0.001
Age				
≤ 60 years	205	16 (7.8)		
> 60 years	160	4 (2.5)	7.403	0.007
Lymph node level				
II/III	226	12 (5.3)		
IV	139	8 (5.8)	0.823	0.346
Pathological type				
Squamous cell carcinoma	192	13 (6.8)		
Adenocarcinoma	118	5 (4.2)		
Other types	55	2 (3.6)	6.802	0.032
Pathological grade				
Highly differentiated	204	0 (0)		
Moderately differentiated	9	2 (22.2)		
Poorly differentiated	152	10 (6.5)	6.594	0.086
Involved nodal stage				
N1/2	355	18 (5.1)		
N3	10	2 (20.0)	6.298	0.043
Surgery				
Yes	205	6 (2.9)		
No	160	14 (8.8)	9.839	0.002
Radiotherapy				
Yes	55	2 (3.6)		
No	310	18 (5.8)	0.425	0.750
Chemotherapy				
Yes	171	6 (3.5)		
No	194	14 (7.2)	2.412	0.166

**Fig. 1:**
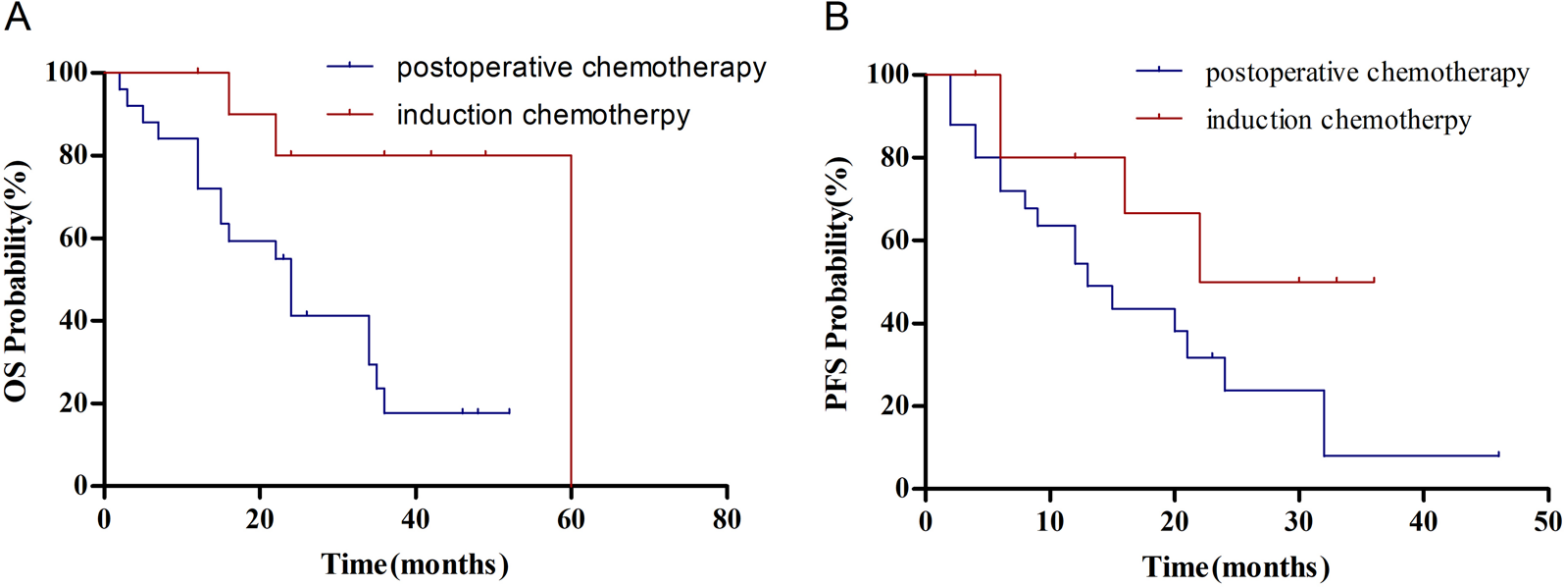
Chemotherapy affects survival of the distant metastasis group for: **A**, overall survival and **B**, disease free survival

Eighty-one (22.2%) patients presented with distant metastasis, including two cases of brain metastases, 12 of bone metastases, 16 of lung metastasis, 20 of abdominal lymph node metastasis, 21 of axillary metastasis, and 40 of mediastinal metastasis. The incidence of metastasis may be higher in patients with adenocarcinoma (*χ*^2^ = 16.635, *P* < 0.001), poorly differentiated lymph nodes (*χ*^2^ = 5.057, *P* = 0.029), and advanced nodal stage (*χ*^2^ = 9.295, *P* = 0.003). In patients with distant metastasis, the poorly differentiated ones accounted for 97.6% (40/41) and the mortality rate was 45% (18/22). In addition, treatment type had a significant effect on the prognosis of patients with distant metastasis (*χ*^2^ = 4.744, *P* = 0.029). Patients treated with induction chemotherapy had better OS (*χ*^2^ = 8.103, *P* = 0.004), but not PFS (*χ*^2^ = 3.356, *P* = 0.067)—than those who received postoperative chemotherapy (Fig. 1A, B). However, postoperative radiotherapy had no remarkable influence on the prognosis of these patients (*χ*^2^ = 0.188, *P* = 0.665).

### Treatment outcomes

The 2- and 5-year overall survival rates of patients were 77.0% and 33.4%, respectively, and the median survival time was 45 months. Kaplan–Meier analysis showed that age, nodal status, treatment, or distant metastasis affected prognosis (Table 3), while Cox multivariate analysis showed that pathological grade, treatment, and distant metastases were independent prognostic factors (Table 4). However, no significant differences were observed in the survival rate of patients in terms of gender, age, pathological type, or involved lymph node level. Local recurrence had an insignificant influence on OS (*P* = 0.065); however, this effect turned to be significant in the multivariate analysis (*P* = 0.047).

**Table 3:** Univariate analysis of the prognosis of patients

Features	No. of patient	OS	PFS
		*P* value	*P* value
Gender			
Male	231		
Female	134	0.631	0.661
Age			
≤ 60 years	205		
> 60 years	160	0.001	0.002
Smoking			
None	224		
No more than 20 years and 20/day	40		
Above 20 years or 20/day	101	0.704	0.667
Alcohol			
None	300		
No more than 20 years and 250 g/day	39		
Above 20 years or above 250g/day	26	0.278	0.281
Lymph node level			
II/III	226		
IV	139	0.546	0.564
Pathological type			
Squamous cell carcinoma	192		
Adenocarcinoma	118		
Other types	55	0.833	0.750
Pathological grade			
Highly differentiated	204		
Moderately differentiated	9		
Poorly differentiated	152	0.001	0.001
Involved nodal stage			
N1	38		
N2a	22		
N2b	185		
N2c	110		
N3	10	0.029	0.025
Initial treatment			
No treatment	54		
Chemotherapy	91		
Surgery	192		
Radiotherapy	28	0.037	0.017
Local recurrence			
Yes	20		
No	345	0.065	0.051
Distant metastasis			
Yes	81		
No	284	0.001	0.001

**Table 4:** Multivariate regression analysis of the prognosis of patients

Multivariate regression analysis (*N* = 395)	OS		PFS	
	HR (95%CI)	*P* value	HR (95%CI)	*P* value
Age	2.442 (0.941–6.342)	0.067	2.228 (0.863–5.750)	0.098
Pathological type	0.786 (0.599–1.031)	0.082	0.778 (0.592–1.023)	0.073
Pathological grade	0.317 (0.148–0.680)	0.003	0.347 (0.162–0.743)	0.006
Initial treatment	0.723 (0.562–0.931)	0.012	0.862 (0.750–0.991)	0.037
Involved nodal stage	0.351 (0.093–1.328)	0.123	0.292 (0.077–1.102)	0.069
Local recurrence	0.442 (0.198–0.988)	0.047	0.475 (0.213–1.063)	0.070
Distant metastasis	3.686 (2.419–5.618)	0.001	4.804 (3.115–7.410)	0.001

**Fig. 2:**
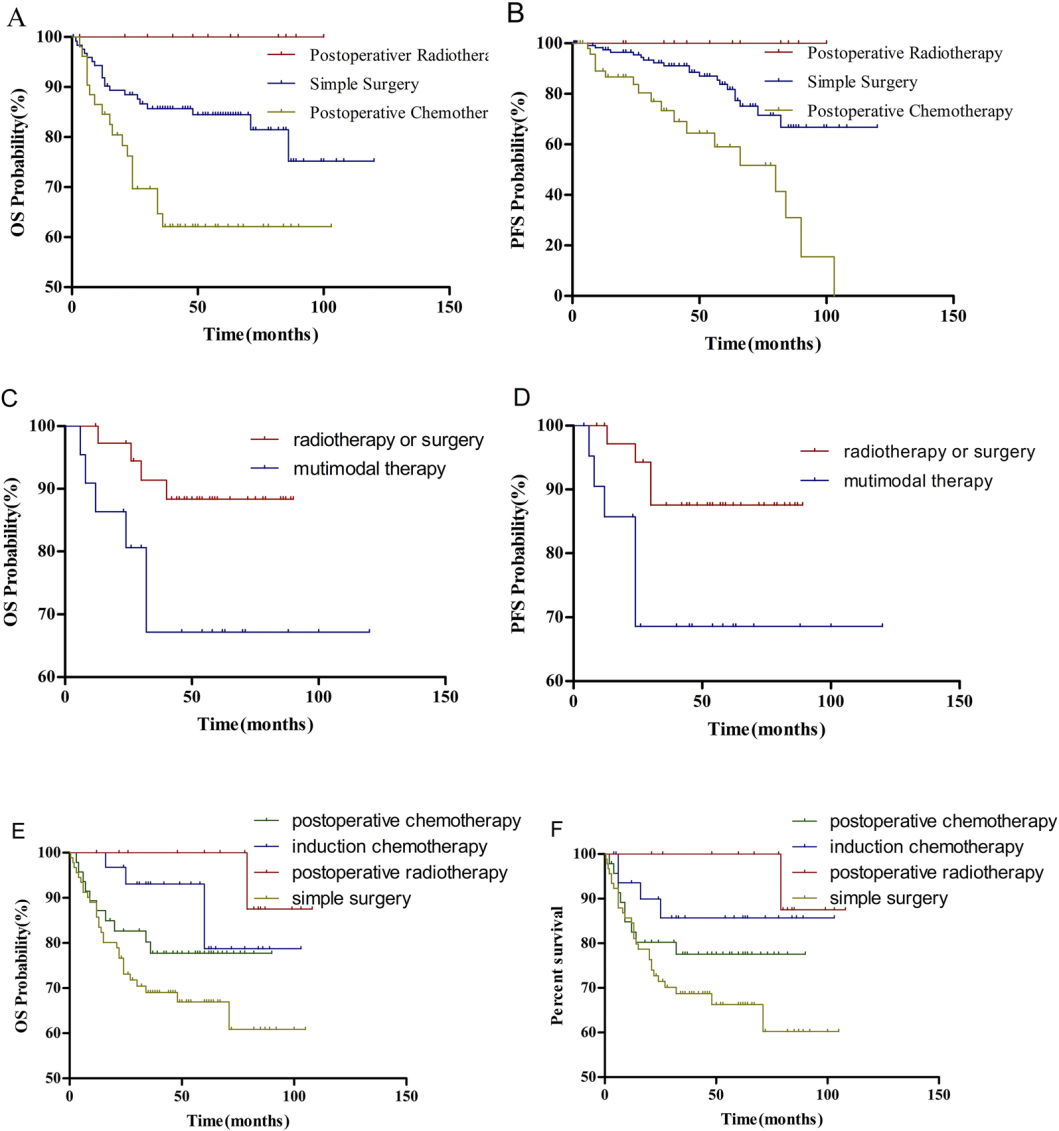
Kaplan–Meier estimates of survival for all patients for: **A**, **C**, **E**, overall survival and **B**, **D**, **F**, disease free survival

As shown in Table 3, initial treatment could significantly affect the prognosis of cervical lymph node metastasis from unknown primary sites. Thus, we assessed the correlation between various treatment strategies and prognoses further to find individual treatments. The results showed that surgery alone had more advantages than other therapies (OS, *χ*^2^ = 12.337, *P* = 0.030). Of the 192 patients who underwent surgery, 126 underwent simple surgery, including 24 patients with N1, nine with N2a, 56 with N2b, 34 with N2c, and three with N3 disease. Moreover, 52 patients underwent surgery followed by chemotherapy and the remaining 14 patients underwent subsequent radiotherapy. Patients who underwent simple surgery or postoperative radiotherapy had better prognoses (OS, *χ*^2^ = 12.336, *P* = 0.002 PFS; *χ*^2^ = 13.604, *P* = 0.001; Fig. 2A, B).

In N1–N2a cancer, the patients who underwent surgery or radiotherapy alone had better survival results than those who underwent surgery combined with chemotherapy or radiotherapy (OS, *χ*^2^ = 4.070, *P* = 0.044; PFS, *χ*^2^ = 3.916, *P* = 0.048; Fig. 2C, D). Among the 91 patients who were treated with chemotherapy as their initial treatment, 54 received simple chemotherapy with advanced disease. Ten and 27 patients eventually underwent surgery and radiotherapy, respectively. Moreover, four patients presented with N1 or N2a cancer, 41 with N2b, 37 with N2c, and nine with N3. For N2b disease or higher nodal status, the significant differences of prognosis were observed between patients who underwent simple surgery (*n* = 93), postoperative therapy (*n* = 61), and induction chemotherapy (*n* = 33). Those who underwent induction chemotherapy and postoperative radiotherapy had a better prognosis when compared with other treatments (OS, *χ*^2^ = 9.515, *P* = 0.023; PFS, *χ*^2^ = 8.173, *P* = 0.043, respectively; Fig. 2E, F).

Of the 192 patients who underwent surgery, 102 underwent selective neck dissection, 61 underwent modified radical neck dissection, and 29 underwent local mass resection. In contrast to partial mass resection, cervical lymph node dissection did not improve prognoses significantly (OS, *χ*^2^ = 2.870, *P* = 0.090; PFS, *χ*^2^ = 3.497, *P* = 0.061).

## Discussion

Various tumors can metastasize to the sites of cervical lymph nodes, complicating the diagnosis and treatment of cervical lymph node metastasis from unknown primary sites. This retrospective study summarized the effects of different treatment regimens on prognosis to provide a guide for clinical treatments. Cervical lymph node metastasis from unknown primary sites is common in men aged 55–65 years [2]. Approximately 40% of the initial symptoms were painless masses [8]; 30–50% of lesions metastasized to levels II and I/III and 10–20% to levels IV/V [8]. The local control ratio approximates to 37–91%, and the 5-year survival rate ranged from 16% to 81% [1]. In this single-center study, we showed that pathological grade, treatment models, and distant metastasis had critical effects on prognosis. These data were similar to the results of previous studies [9].

Treatments of occult carcinoma metastatic to cervical lymph nodes remain controversial [3]; however, overwhelming evidence has shown that neck metastasis is the focus of clinical treatment [10]. Studies have shown that treatment options must be developed according to lymph node metastasis sites, nodal status, and pathological type [8]. Our further analysis of treatments limited to different nodal status indicated that simple radiotherapy or surgery is superior to systemic combination therapy at the early stage. Interestingly, there was no obvious difference in prognosis between patients who underwent simple surgery and radiotherapy.

Patients with locoregional failure may have more aggressive cancer. In our series, the younger individuals generally had better prognoses than the older ones; however, they had no advantages in terms of local recurrence. Notably, local recurrence rate was significantly associated with OS. This effect was not obvious in the univariate analysis, partly because local failure usually indicates a high probability of distant metastasis with interaction influence, as proposed by a previous study [11].

Advancements in radiation techniques and surgery have changed the failure pattern for patients with distant metastasis [12]. Most patients with distant metastases had a poor prognosis; however, previous studies have not systematically analyzed the treatments of distant metastasis [13]. We found that induction chemotherapy alongside surgery or radiotherapy improved long-term survival, which may be partly caused by a decrease in the risk of developing remote spread.

Radiotherapy has an important role in the preservation of cervical function and treatments of potentially hidden primary tumors, except the elimination of primary tumors [14]. However, whether patients should receive double-sided radiation remains controversial. Chen et al. [15] have reported that selective irradiation of the ipsilateral oropharynx and neck is more effective for local control in patients with cervical lymph nodes from occult squamous carcinoma with p16-positive. Bilateral radiotherapy showed no advantages with respect to radiation-induced side effects and outcome [16]. In contrast, several studies have shown that intensity-modulated radiation therapy to both sides of the neck and the mucosal site leads to improvements in local control and survival [7]. Hence, these views require in-depth assessments in future. In addition, surgery is significantly important for this disease due to the side-effects of radiotherapy and postoperative pathology which can be used as a basis for subsequent treatment [8].

Postoperative radiotherapy had limited benefits, particularly in patients with distant metastases. Patients who receive induction chemotherapy and postoperative radiotherapy may have better survival; some studies have reported that chemotherapy is an integral part of the standard non-surgical treatment for locally advanced head and neck cancer [17, 18]. Chemotherapy may have survival benefits; however, it remains to be associated with acute toxicity [4] and the application of induction chemotherapy is controversial [19]. Our findings suggested that induction chemotherapy alongside surgery or radiotherapy is more advantageous than single surgery or radiotherapy, particularly in advanced disease. As a consequently, we recommend induction chemotherapy for patients with advanced disease who are more likely to have a distant transfer. These findings were supported by previous studies which argue that chemotherapy can improve the local control rate and reduce the risk of distant metastasis [7]; therefore, it can relieve symptoms of advanced malignancy.

Clinically, the highest probability of detecting primary lesions is approximately 62% [20]. A series of studies have attempted to explore primary lesions of human papilloma virus (HPV)-associated cervical squamous metastases from an unknown primary [21, 22]. Park et al. [23] have shown a sensitivity and negative predictive value of 90% and 93%, respectively, for HPV in the detection of metastasis in the oropharyngeal primary site. Although there have been many attempts, the low gravity with which primaries are detected impedes the optimal management of primary lesions. Coster et al. [24] have reported the clinical results of curative resection via neck dissection or excisional biopsy alone. They conclude that patients with N1 cancer without extracapsular extension (ECE) can be managed by surgery alone, whereas those with advanced disease and/or ECE are candidates for postoperative adjuvant radiation therapy. These findings are supported by a study by Yamazaki [25], which has reported negative ECE status is a factor associated with favorable OS and PFS. ECE is an unfavorable prognostic factor of neck recurrence, cause-specific survival, and overall survival.

Clinical research data about cervical lymph node metastasis from unknown primary sites are limited [26]. Based on the correlation between various treatment options and prognoses, we propose approaches that may be beneficial to survival. However, this research has limitations due to its retrospective nature and inability to adequately detect HPV status. More prospective clinical trials must be conducted to identify more effective treatment options.

## Conclusions

Pathological grade, treatments, and distant metastasis influenced the prognosis of cervical lymph node metastasis from unknown primary sites. Furthermore, local recurrence were independent risk factors for OS. Simple surgery or radiotherapy for nodal status was recommended for N1–N2b cancer. Induction chemotherapy or surgery alongside radiotherapy improved the prognosis of patients with N2b–N3 cancer. Meanwhile, patients who were at high risk of developing distant metastasis should receive induction chemotherapy.

## Data Availability

Not applicable.
